# A Comparative NLP-Based Study on the Current Trends and Future Directions in COVID-19 Research

**DOI:** 10.1109/ACCESS.2021.3082108

**Published:** 2021-05-20

**Authors:** Priyankar Bose, Satyaki Roy, Preetam Ghosh

**Affiliations:** 1 Department of Computer ScienceVirginia Commonwealth University 6889 Richmond VA 23284 USA; 2 Department of GeneticsUniversity of North Carolina at Chapel Hill 2331 Chapel Hill NC 27515 USA

**Keywords:** COVID-19, natural language processing, coefficient of variation, mean squared error

## Abstract

COVID-19 is a global health crisis that has altered human life and still promises to create ripples of death and destruction in its wake. The sea of scientific literature published over a short time-span to understand and mitigate this global phenomenon necessitates concerted efforts to organize our findings and focus on the unexplored facets of the disease. In this work, we applied natural language processing (NLP) based approaches on scientific literature published on COVID-19 to infer significant keywords that have contributed to our social, economic, demographic, psychological, epidemiological, clinical, and medical understanding of this pandemic. We identify key terms appearing in COVID literature that vary in representation when compared to other virus-borne diseases such as MERS, Ebola, and Influenza. We also identify countries, topics, and research articles that demonstrate that the scientific community is still reacting to the short-term threats such as transmissibility, health risks, treatment plans, and public policies, underpinning the need for collective international efforts towards long-term immunization and drug-related challenges. Furthermore, our study highlights several long-term research directions that are urgently needed for COVID-19 such as: global collaboration to create international open-access data repositories, policymaking to curb future outbreaks, psychological repercussions of COVID-19, vaccine development for SARS-CoV-2 variants and their long-term efficacy studies, and mental health issues in both children and elderly.

## I. Introduction

Many virus-borne diseases like Ebola, Influenza, and now COVID, have threatened mankind. Amongst these, variants of the coronavirus have caused global pandemics, such as MERS, SARS, and COVID-19, by mainly manifesting as respiratory infections in humans [1]–[3]. The coronavirus is an RNA virus that pushed the world to a socio-cultural and economic standstill. It has also inspired scientific research in many different domains beyond the realm of medicine. Understandably, scientific literature on COVID-19 skyrocketed after January 2020 and keeping track has become a challenge, especially when the body of literature on existing diseases like Influenza, Ebola, MERS is still growing. Over the past two decades, more than 35 million articles on virus-borne diseases have been published [4]. A comparative analyses on how the scientific practices and findings differ for COVID-19 with respect to other virus-borne diseases may shed light on the similarities and dissimilarities between these virus-borne diseases, and identify top strengths and shortcomings in clinical methods, practices, and treatment besides also innovations in public policies, for each disease. Thus analyzing textual information of published articles in these areas can highlight research advances in virus-borne diseases in general, and COVID-19 in particular, and inform future directions on scientific research, clinical trials, course of treatment, socioeconomic implications and administrative decision-making.

### A. Background

Enormous efforts have gone into COVID-19 research within a short span of time. Research works on COVID-19 symptoms [5], [6] and screening [7], [8] were particularly conducted, including techniques like telephone based screening. COVID-19 testing [9], [10] and its spread [11], [12] have also been a very popular research direction. Medications for COVID-19 [12], [13] have been addressed in some works which can be helpful in drug discovery. COVID-19 vaccination has emerged as a very important topic where substantial research has been conducted [14], [15]. A plethora of research papers discuss the impact of the pandemic on mental health [16]–[20]. The socioeconomic, political and cultural aspects of COVID-19 outbreak [21], [22] have also been extensively covered in prior research; these aspects were analyzed in [11], [23]–[25] to study the effects of lockdown, social distancing and US job losses in different sectors. Specifically, in [24], the authors used topic modeling to analyze the effect of COVID-19 in the job sector. Artificial intelligence and machine learning approaches for building predictive models on COVID-19 data were reviewed in [26], [27], while machine learning and deep learning methods on noisy chest X-ray images [28] were employed to detect COVID-19 in [29]–[31]; data augmentation techniques for both X-ray and CT image data [32] were also investigated [31].

There have also been some recent efforts in textual analysis on COVID-19 related research articles. Analysis of bibliometric aspects of the studies and a scoping analysis led to the identification of the main safety-related topics in COVID-19 [33]. Similarly, bibliometric analysis on COVID-19 related articles was accomplished using text mining approaches [34]–[36]. Textual analysis of social media data has the potential to study public perceptions, attitudes and trends related to COVID-19 [42]–[44]. The topics, key terms, and features of the COVID-19 tweets were also analyzed using Topic Modeling, UMAP, and DiGraphs [37]. Text mining techniques have been extremely popular for exploratory analysis on coronavirus-related research. Although a considerable amount of such exploratory analysis is only related to COVID-19, a number of research works have also studied the coronavirus in general. A bibliometric analysis of 395 journal articles from the field of social sciences related to coronavirus was performed by using the ‘biblioshiny’ package [38]. Text mining techniques like topic modeling using LDA have been widely used to extract the research hotspots and other related information from the articles on different coronavirus-related diseases such as COVID-19, SARS, and MERS, etc. [40], [41]. Also, interesting recurring patterns have been identified by using scientometric comparisons across various coronavirus literature [39]. The different areas of related works and the key-takeaway from them are shown in Table 1. In these works, the authors have considered the bibliometric data of articles to perform their analysis.

**TABLE 1:** Summary of the Different Facets of COVID-19 Research

Area	Dataset	Methods	Key Takeaways
COVID-19 detection [29]–[31]	Combined COVID-19 Dataset [32] and Noisy COVID-19 X-ray Dataset [28]	Machine Learning and Deep Learning models	LWL-SOM performed better than the current machine learning models on this data
Bibiliometric analysis of COVID-19 [33]–[36]	COVID-19 bibliometric data	Packages like VOSviewer, Bibliometrix and R software	Identified some research themes but unable to address other themes like the long-term impact
Textual analysis of COVID-19 tweets [37]	COVID-19 tweet data	Topic Modeling, UMAP, and DiGraphs	Identified the topics, key terms, and features of COVID-19 tweets
Comparative analysis of COVID-19 research with respect to other coronavirus research [33], [38], [39]	SARS, MERS and COVID-19 publications dataset	Packages like VOSviewer, biblioshiny and R software	Identified the influential aspects of different areas in coronavirus research
Topic Modeling: COVID-19 research with respect to other coronavirus research [40], [41]	SARS, MERS, COVID-19 and other CoV publications (CORD-19) dataset	Latent Dirichlet Allocation	Identified inter-relationships between various coronavirus research to address knowledge gaps

In this paper, however, we compare the abstracts on other virus-borne diseases like MERS, Ebola, and Influenza against that of COVID-19 related studies for the first time. The objective of this work is to report and analyze the implications of the semantic and statistically significant words in COVID-19 research compared to those on MERS, Ebola and Influenza. Our goal is to identify the research gaps in COVID-19 that can motivate future research. We discuss the salient contributions of this study next.

### B. Contributions

**FIGURE 1.**
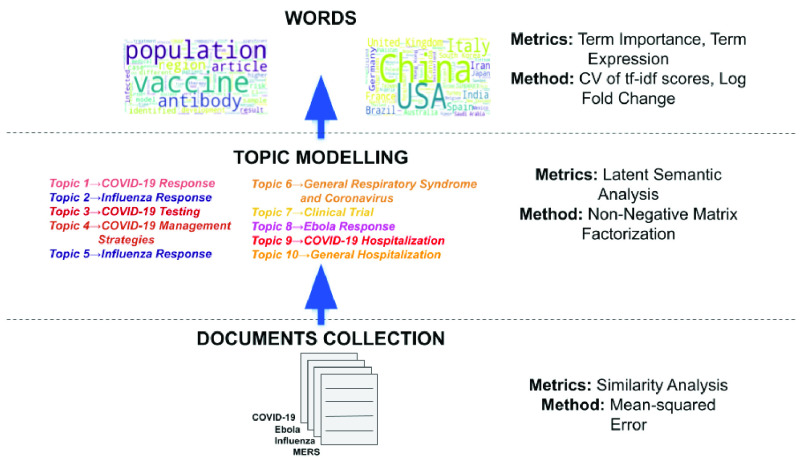
Summary of contributions: Each document consists of the abstracts of scientific literature of each disease used to analyze significant over- and under-expressed words, topics, countries and research articles.

We apply natural language processing to carry out a comprehensive textual analysis on COVID-19 publications. We infer keywords, topics, countries, and research articles, etc., which yield insights into the present challenges of COVID research from a social, economic, demographic, psychological, epidemiological, clinical, and medical standpoint. A schematic diagram of our workflow is illustrated in Figure 1. The contributions of the research are summarized as follows:

• Using the concept of *coefficient of variation*, we report the significant words present in the abstracts of the articles on three diseases apart from COVID-19, namely, MERS, Ebola, and Influenza. We identify the common, under-expressed, and over-expressed words in the abstracts of the COVID-19 articles with respect to those of Influenza, MERS, and Ebola.
• We quantify the similarity between each pair of diseases (in terms of *mean squared error* as a measure) and discuss the possible context behind the significant words identified in this study. We identify relevant countries, topics, and research articles that show the timeliness of COVID-19 research as well as their limitations.
• Our analysis throws up keywords such as *healthcare*, *clinical*, *risk*, *morbidity* that suggest that the scientific community are responding to the immediate existential threats related to transmissibility, health risks, and clinical care and are not yet invested on the long-term immunization and drug-related solutions. While research on medications related to COVID-19 emerges as a popular subject of interest, our study underscores the importance of diagnostics, containment, and short-term treatment plans and the need to broaden the scope of exploration.
• We report the names of the top countries, like China, USA, Italy, UK, and India, that come up in COVID-related research abstracts and show how mentions of such country names in the scientific literature have evolved over time.
• We also report the top topics in the research abstracts on COVID-19, MERS, Ebola, and Influenza based on their association with the top keywords.

## II. Approach

In this section, we outline the different statistical and natural language processing methods applied during the textual analysis of the scientific literature on virus-borne diseases. We depict in Figure 1 that our analysis involves the identification of significant words in COVID-19 literature using metrics like coefficient of variation (CV), fold change, etc. We then apply latent semantic analysis to identify topics comprising these words. Finally, we study the similarities in the scientific literature of MERS, Ebola, Influenza and COVID-19.

### A. Data Collection

The data was collected from Pubmed [45] which is an open-access search engine by the National Center for Biotechnology Information (NCBI) to retrieve the MEDLINE database of abstracts and citations on biological and biomedical topics. For each of the four virus-borne diseases like COVID-19, Influenza, MERS, and Ebola, we collected the abstract data of the research papers in PubMed under ten different topics that address the genetic, epidemiological, clinical, psychological, socioeconomic, and public policy related questions about the corresponding disease. For example, in the case of COVID-19, the ten topics under which we have collected the abstract data are (1) COVID-19, (2) COVID-19 policy, (3) COVID-19 vaccine, (4) COVID-19 epidemiology, (5) COVID-19 genomics, (6) COVID-19 comorbidity, (7) COVID-19 outcome, (8) COVID-19 economy, (9) COVID-19 factors and (10) COVID-19 society. We restricted ourselves to the top 10, 000 abstracts for each of the topics. For topics not meeting this threshold, all the available instances were considered. In order to facilitate the comparative analysis, we created an integrated dataset (1) constituting the literature of the diseases and (2) COVID vs non-COVID abstract data.

### B. Data Preprocessing

The textual PubMed abstract data from these diseases contain a lot of noise consisting of bibliographic details. Irrelevant information like journal identification number, author information, and non-English words were directly removed from the text using regular expressions. The stopwords were eliminated using the gensim platform [46] in Python. Finally, *lemmatization* was performed to group inflected forms of the same words so they could be analyzed as a single item.

### C. Over- and Under-Expressed Words

**FIGURE 2.**
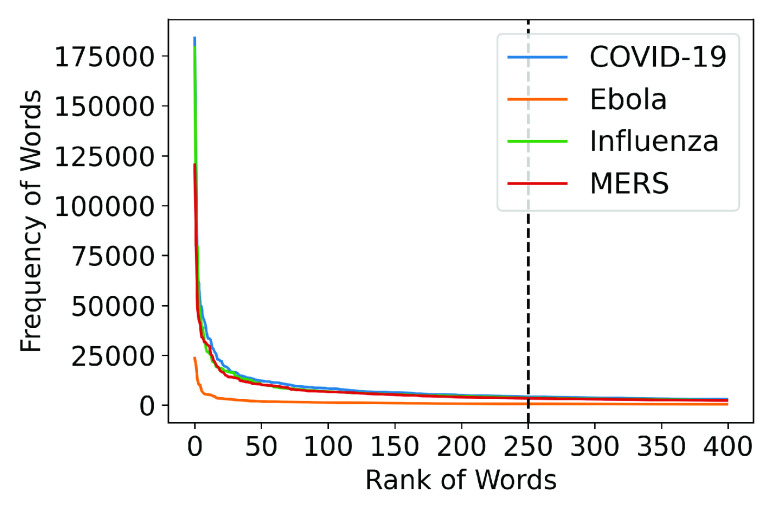
Frequency of the top 400 words in the Research Abstracts.

We estimated the over- and under-expressed words (defined in Sec. II-C) in the COVID-19 abstracts in comparison to the research abstracts of the other diseases. Note that the expression of a word is a relative measure of its occurrence. This analysis was achieved in the following four steps:

• **Important words in each disease document:** The 250 most frequent words were selected for each disease from the preprocessed data on each disease. The 250 most frequent words were chosen because they provide a comprehensive representation of the popular words in the dataset. Frequency distribution of the top 400 words in the research abstracts of the four diseases, is shown in Figure 2 and depicts a very steep decline in the frequencies till the top 50 words and then, a gradual decline in the frequencies till the top 150 words. After that, the decline in frequency is negligible and the curve starts to flatten. Hence, we went ahead with the top 250 words as it allowed us to consider 100 more words after the curve starts to flatten; after 250 words, the curve straightens further. The range of the frequencies of the top 250 words in each document is in a similar range for COVID-19 and Influenza, and MERS to some extent too but in the case of Ebola, the range of the frequencies is quite smaller. The frequency of the words varied roughly between more than 100K and a few thousand for COVID-19, Influenza, and MERS whereas it varied between a little more than 23K and a few hundreds in case of Ebola.
• **Significant common Words:**
  We use latent semantic analysis to identify significant words across all diseases, generated from the top 250 most frequent words of each disease. The relative importance of common words is measured in terms of the *tf-idf* score. Specifically, term frequency-inverse document frequency (*tf-idf*) is a statistical measure for calculating the importance of a term to a document in a collection of documents [47]. Term frequency (*tf*) of a word $t$ is generally calculated by taking the ratio of the number of times a word appears in a document $d$ (denoted by $f_{t,d}$) to the sum of the frequency of all the terms in $d$. Inverse document frequency (*idf*) quantifies how rare a word is in the corpus of documents $D$, by calculating the logarithm of the ratio of the total number of the documents and the number of documents that contain the word. The *tf-idf* score is calculated as:
  \begin{align*} tf(t,d)=&\frac {f_{t,d}}{\sum _{t' \epsilon d} f_{t',d}}\tag{1}\\ idf(t,d,D)=&log \frac {N}{|{d \epsilon D: t \epsilon d}|}\tag{2}\\ tf - idf(t,d,D)=&tf(t,d) \times idf(t,D)\tag{3}\end{align*}
  Following this, we measure the coefficient of variation (CV) of each word as the ratio of the standard deviation ($\sigma $) to the mean ($\mu $) of the *tf-idf* scores of these words across all documents (i.e., $\frac {\sigma }{\mu }$). Thus, low CV indicates a lower standard deviation and higher mean. In other words, it is a combination of a higher mean occurrence of a word and a low deviation from this mean across documents. Note that there exist words with equal *tf-idf* across the four diseases. Although mathematically their CV is infinite, we have considered these words to have $CV = 0$. Since CV cannot be negative as both mean and standard deviation are positive values, the lowest value of CV is 0. In our context, the overall importance of a term is inversely proportional to its CV i.e., the lower the CV, the higher is the importance of a word.
• **Similarity across diseases:** To understand how the scientific literature vary across any pair of diseases $i$ and $j$, we calculate the mean squared error $MSE (L_{i}, L_{j})$. Here $L_{i}$ and $L_{j}$ are the vectors containing *tf-idf* scores of the common words (refer Sec. II-C) for diseases $i$ and $j$, respectively, arranged in the lexicographical order.
• **Over-expressed and under-expressed words:** The concept of log fold change is used to measure the quantitative change in an observed phenomenon in a given scenario with respect to a control scenario [48]. For any word $w$, $LR(w) > 0$ and $LR(w) < 0$ represent over- and under-expressed words, capturing the extent of relative increase or decrease in the occurrence of a word in COVID-19 literature vis-à-vis all the four diseases considered in this study. Given the frequency of word $w$, $h_{d}(w)$ in disease type $d$, it is measured as:
  \begin{equation*} LR(w) = \log _{2} \frac {h_{covid}(w) + 1}{ \frac {1}{d} \times \sum _{d} ({h_{d}(w) + 1})}\tag{4}\end{equation*}

### D. Relevant Topics

Let us consider a matrix of $n$ documents and $m$ significant common words $[X]_{n*m}$. We apply non-negative matrix factorization to identify two sub-matrices $[W]_{n*d}$ and $[H]_{d*m}$, i.e., $[X]_{n*m} \sim [W]_{n*l} \times [H]_{l*m}$, where $l$ denotes a latent factor. Non-negative matrix factorization has been a prominent technique for information retrieval with huge applications in computational neuroscience, multidimensional data analysis, etc. [49]. The $W$ and $H$ matrices acquired by this decomposition allow us to utilize the latent factors (as topics) to capture the relationship between any pair of documents as well as the words. Also, we infer the semantic significance of a topic by analyzing the words with the highest weights in that topic.

### E. Top Research Countries and Articles

We termed the countries registering the highest occurrence across COVID-19 abstracts as top countries featuring in COVID-19-related research. Identification of top countries involved pruning the COVID-19 abstracts with variants in the country names. For instance, we encountered *United Kingdom*, *UK*, *England* – all of which refer to England. Similarly, the significance of a research article in the COVID-19 document is calculated in terms of the presence of the most important common words in the title or contents of the abstracts.

## III. Results

The results follow the same organization as the schematic depicted in Figure 1. We first analyze significant words (including countries) in terms of over- or under-expression in COVID-19 literature. Following this, we identify key topics and analyze the textual similarity in the literature of the different diseases.

### A. Important Words

**FIGURE 3.**
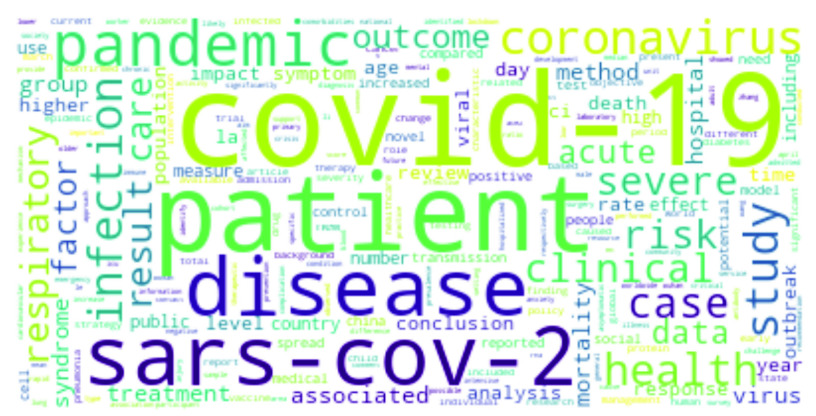
Most frequent words in COVID-19 research.

**FIGURE 4.**
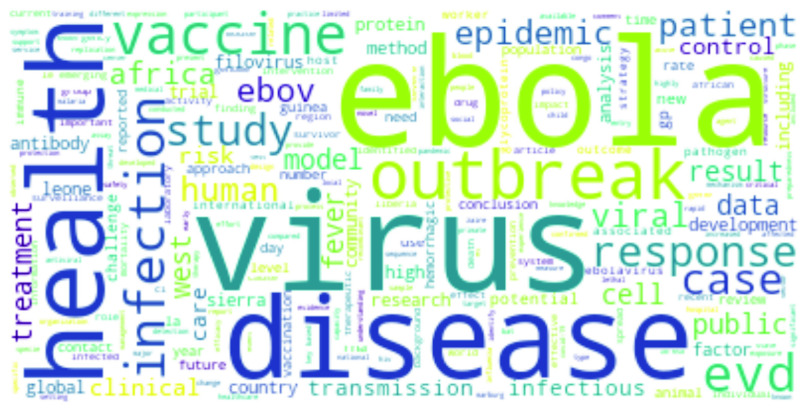
Most frequent words in Ebola research.

**FIGURE 5.**
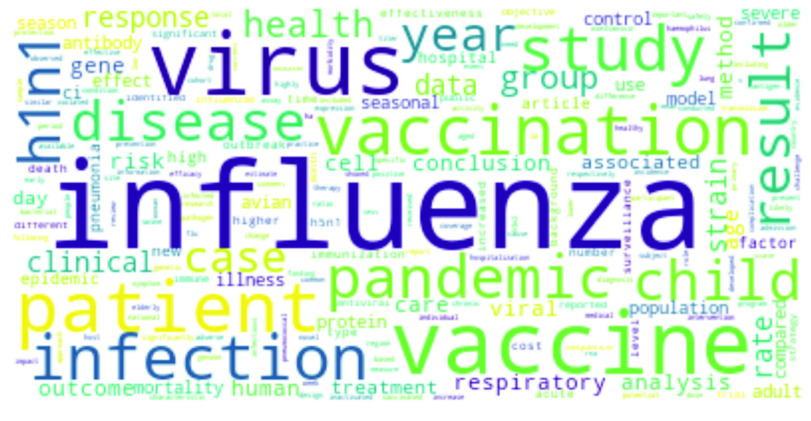
Most frequent words in Influenza research.

**FIGURE 6.**
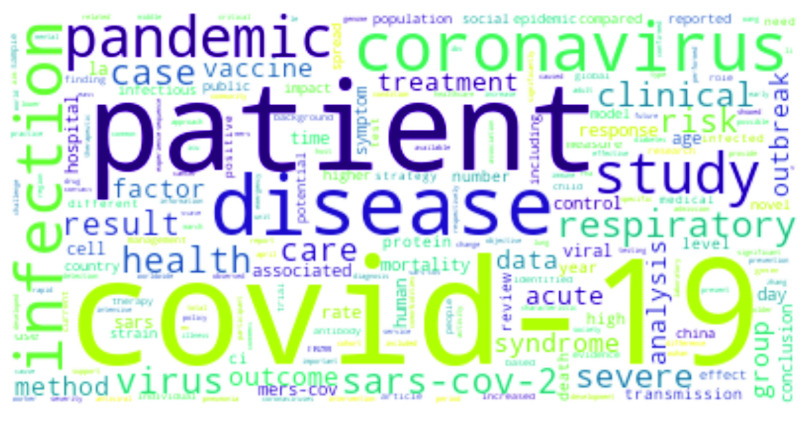
Most frequent words in MERS research.

The data from abstracts in COVID-19 and other viral disease-related research is preprocessed to filter out the noise and focus on the important words. The top 250 words are chosen from the abstracts of each disease category. These important words are analyzed and discussed as follows:

• **COVID-19:** Figure 3 shows the top COVID-19 words, where large font size is commensurate with higher term frequency. The virus responsible for the outbreak, *SARS-CoV-2*, is predictably a top word [6], [50]–[52]. Similarly, *Coronavirus*, *respiratory*, *severe* and *acute* are also top-ranked words [53], [54]. Other words like *pandemic*, *transmission*, *outbreak, etc* have often been mentioned [7], [21], [53], [55]. In addition, the city and country of origin of the virus – *Wuhan* and *China* – have come up extensively [51], [53]. Interestingly, studies have been carried out to understand the association between *cancer* and COVID-19 [56]. Such studies deal with cancer in children [57], and breast cancer [58], among others. Apart from cancer, comorbidities like *diabetes* and *cardiovascular* diseases as well as fever as a symptom of COVID have been mentioned [59], [60]. Studies have been also carried out on COVID-19 *vaccine*s and people’s reaction to it [15]. Mental health is another popular topic, since COVID-19 has had serious psychological consequences. This is well reflected in some recent research works that addressed the issue of *mental* healthcare and short- and long-term psychological impacts of the pandemic [17], [18], [61]. Interesting topics like mental health issues in the elderly population or in people with comorbidities [20], [61], individual mental health [16], mental health effects due to COVID-19 media coverage [19], etc. have also been addressed recently.
  *RNA* and *protein* have come up while describing the single-stranded RNA-based COVID-19 virus and the effects of different proteins on it [53]. Studies on pneumonia or COPD (Chronic obstructive pulmonary disease) have been mentioned, making words like *pneumonia*, and *lung* highly relevant [62]. In addition, there are other works assessing the expression patterns and genetic polymorphism of Angiotensin Converting Enzyme 2 or *ACE2* [59], [63] – an enzyme present in the cell membranes in the lungs, arteries, heart, kidney, and intestines, that is responsible for reducing blood pressure. *ACE2* has been mentioned with regard to a drug to treat cardiovascular diseases and serves as an entry point for many coronaviruses including COVID-19.
• **Ebola:** *Ebola* is understandably the most significant term, followed by *virus*, and *disease* [64]–[66]. Terms like *vaccine* [67] and *epidemic* [68] are next in line. Interestingly, the term *pandemic* [69] is less used in case of Ebola than other virus-spread diseases. The words like *West*, *Africa*, *Seirra Leone*, *Guinea*, *Liberia* are mentioned in the context of the first community transmission of the Ebola epidemic in West African countries. Similarly, *Congo*, a place that witnessed Ebola outbreak in a pre-vaccine period, has also been mentioned [67]. Terms *RNA*, *protein*, *VP35* (viral protein 35) and *VP40* (viral protein 40) occur in the abstracts of Ebola research articles, where types of Ebola virus and their medical pathogenesis are discussed [70]–[72]. The symptoms of the disease is widely discussed through words like *hemorrhagic*, *fever* and *bats* [66], [68]. *Ebola* and *Marburg* are a type of *filovirus* and hence these terms appear on Ebola research articles where filovirus were mentioned [65], [73], [74]. Modified Vaccinia virus Ankara (MVA) is mentioned in the context of vaccine development to combat diseases like *Influenza*, *COVID-19*, *HIV*, *malaria*, *Influenza*, and Ebola [69], [75]. Research on SARS-CoV and SARS-CoV-2 also discuss their severity, as indicated through terms like *ventilation* [76] and Intensive Care Unit (*ICU*) [77], [78]. Most frequent words for Ebola related research abstracts are illustrated in Figure 4.
• **Influenza:** Apart from *Influenza*, the terms *virus*, *vaccine* and *patient* get frequent mentions [79], [80]; the virus that is responsible for causing influenza, *H1N1*, as well as a subtype of Influenza A virus, *H3N2* appears often [81]. There are references to genotyping [82], health implications [83] (such as pregnancy [84]) and the *avian* Influenza A virus i.e *H5N1* [85], [86]. The drug used to treat flu or Influenza is known as *oseltamivir*; this antiviral drug often comes up in research abstracts on Influenza virus [87], [88]. As an after-effect of Influenza, the term *pneumonia* [89] is mentioned, while *Haemophilus* becomes relevant as ample research discusses Haemophilus influenzae [90]. *Haemaglutinin* is a membrane glycoprotein on the Influenza virus and quite a few research works talk about this membrane in connection with Influenza [91]. The most frequent words in the Influenza related research abstracts are also depicted in Figure 5.
• **MERS:** Unlike other diseases, the top word in case of MERS is *COVID-19*. Note that the words next in order are *coronavirus* and *pandemic* [92], [93]. Likewise, words like *SARS* and *SARS-CoV* have risen into prominence in the context of MERS research [94]–[96]. *Diabetes* is enlisted among meaningful words, since many studies talk about the effect of diabetes on a coronavirus (SARS, MERS or COVID-19) infected person [97].
  To our surprise, the terms like *MERS* and *MERS-CoV* appear after COVID-19 and SARS. This is because several research articles on MERS have also dealt with other coronaviruses [98], [99], whereas only a few are solely dedicated to MERS [100]. *Cancer* once again appears frequently due to the ample research on cancer patients suffering from the disease [101]. The term *IBV*, short for Infectious Bronchitis Virus [102], [103] come up, while a lot of research on Porcine Epidemic Diarrhea Virus (*PEDV*) [104], [105] is present in the MERS research abstracts. Once again, the most frequent words in the MERS research abstracts are shown in Figure 6.

**FIGURE 7.**
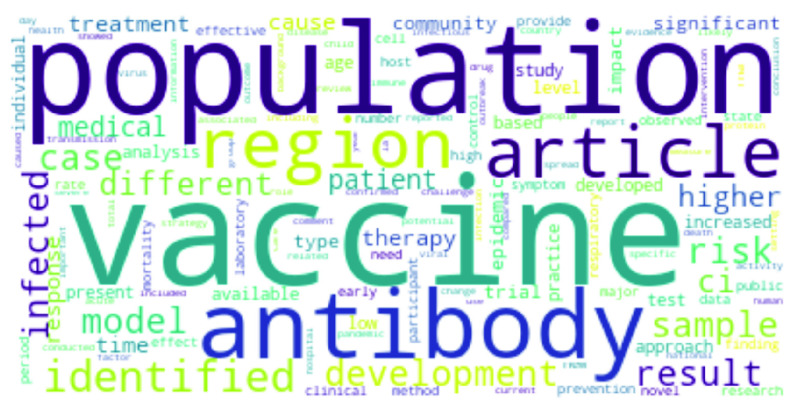
Significant common words across all diseases.

Overall, there is a greater emphasis on the course of transmissibility, and health implications for patients with preexisting conditions in COVID-19 literature. For the other diseases, we observe higher mentions of the treatment measures and their evolution in terms of geographical epidemiology. This shows that we are still in the nascent stages of COVID-19 research with a limited understanding of treatment and mitigation strategies.

### B. Comparison of Term Expression Across Diseases

**FIGURE 8.**
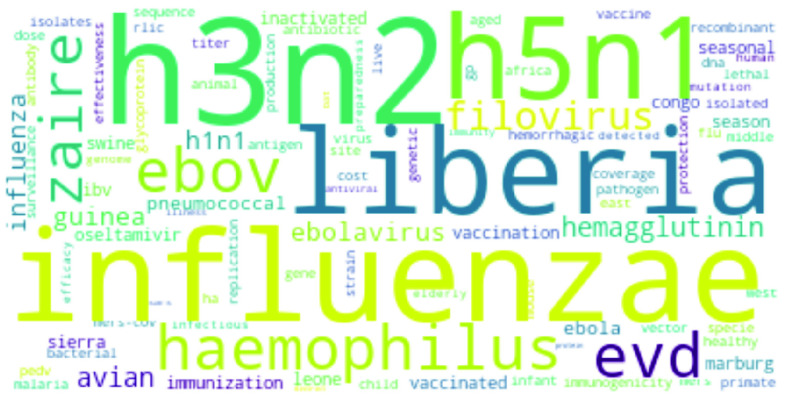
Top under-expressed words based on log fold change.

**FIGURE 9.**
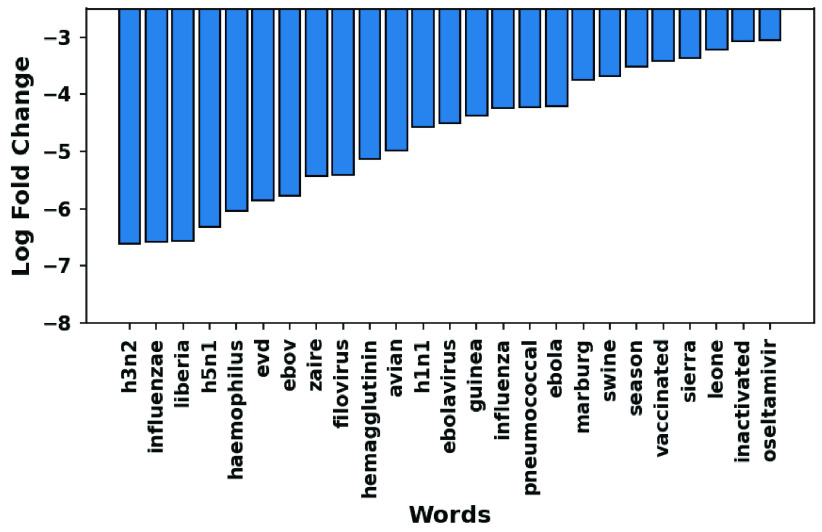
Distribution of the first 25 under-expressed words by the log ratio ($LR$).

In Section II-C, we define over- and under-expressed words as those that exhibit a relative increase and decrease in COVID literature as compared to all the four diseases considered in this study. Figures 8 and 9 visualize the top 25 words showing the highest variation in expression (i.e., log fold change measuring under-expression). It is worth noting the occurrences of Influenza viruses like *H3N2*, *H5N1*, *H1N1*, which have been discussed widely in the context of all virus borne diseases. Similarly, we find the mentions of places like Guinea, Liberia, and Sierra Leone that have suffered large-scale outbreaks of Ebola commonly manifested in flu-like symptoms. Considering the early stages of research and distribution on immunization, the term *vaccination* is relatively underrepresented in COVID literature.

**FIGURE 10.**
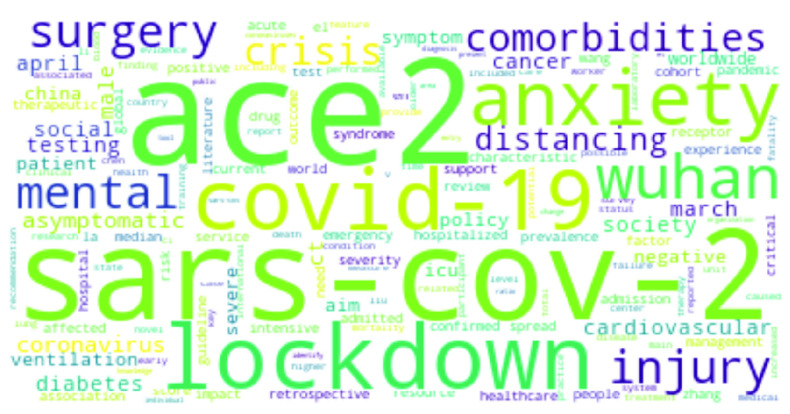
Top over-expressed words based on log fold change.

**FIGURE 11.**
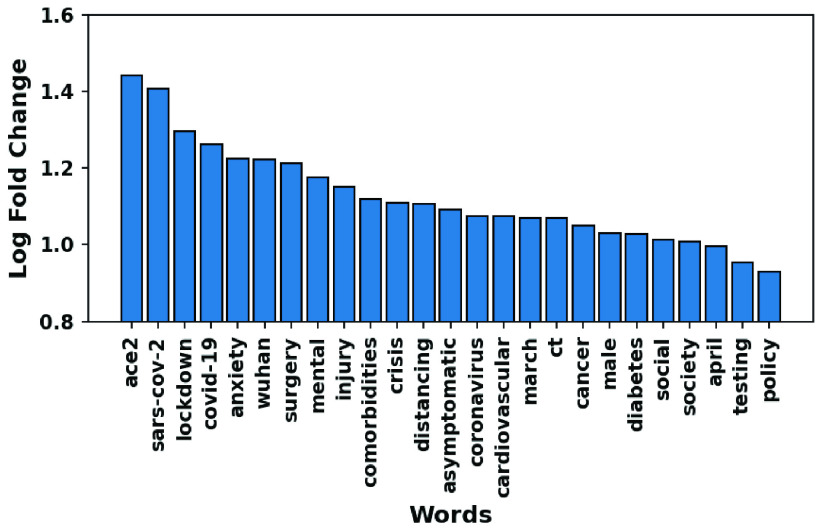
Distribution of the first 25 over-expressed words by the log ratio ($LR$).

Similarly, Figures 10 and 11 show the over-expressed words in COVID-19 abstracts, and the distribution of the first 25 over-expressed words by $LR$ score. Understandably, the most over-expressed words (also flagged as important in Sec. III-G) are *ACE2*, *SARS-CoV-2* and *lockdown*. The relationship between susceptibility to COVID-19 and preexisting conditions is evident where terms such as *cancer*, *diabetes*, *cardiovascular* and *comorbidities* are over-expressed. Observe a more even distribution of the top over-expressed words as compared to the under-represented counterparts. Similarly, we have the list of under-expressed words in Fig. 9. We discuss them in more detail in Section III-G.

### C. Significant Countries

**FIGURE 12.**
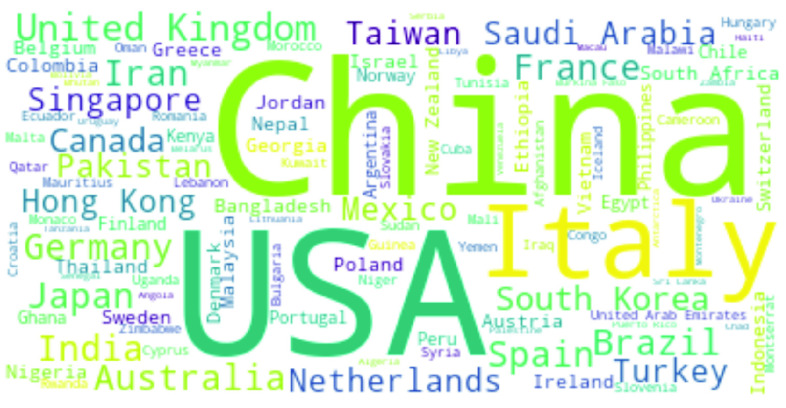
Names of the most mentioned countries in COVID abstracts.

**FIGURE 13.**
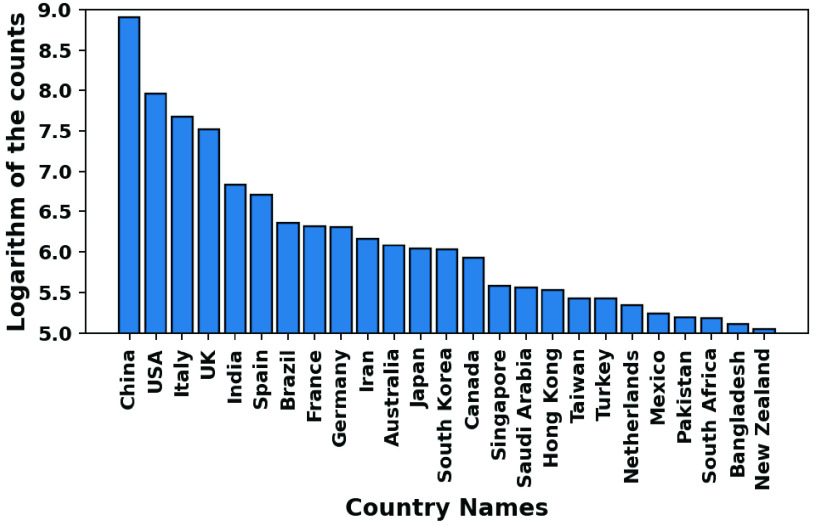
Frequency of 25 most mentioned countries in COVID research.

**FIGURE 14.**
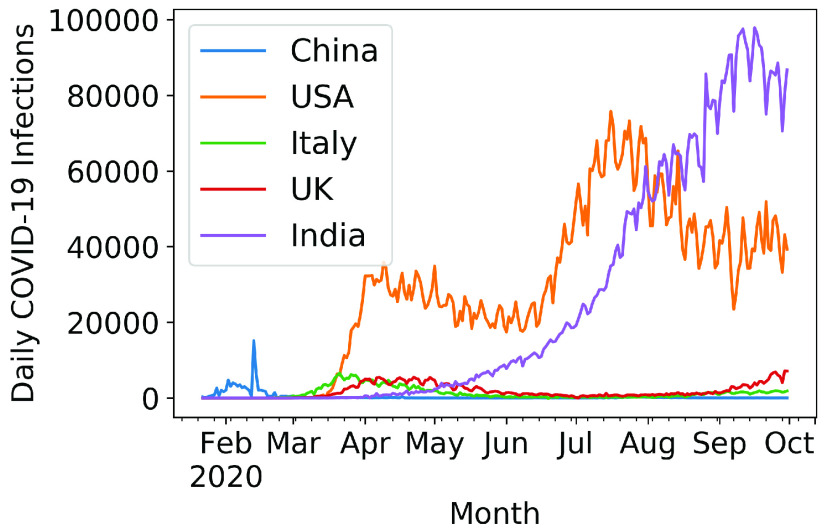
Daily COVID-19 infections in the 5 countries mentioned highly in COVID-19 research abstracts.

**FIGURE 15.**
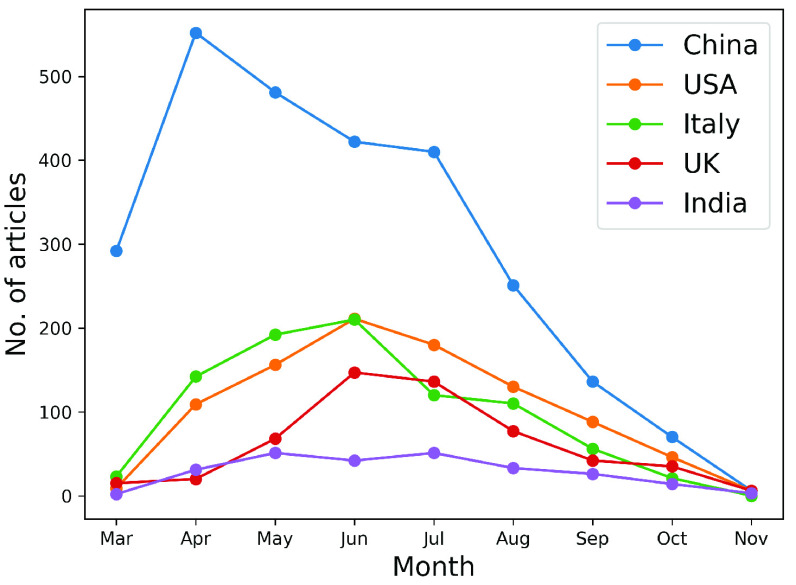
Evolution of the frequency of occurrence of the most mentioned countries in COVID-19 research abstracts.

A significant country is one that appears (as a region where the scientific study on COVID-19 is focused) in abstracts with a high number of significant common words. Figures 12 and 13 show the country names pictorially and log frequency of the words associated with each country, where China, USA, Italy, UK, and India are ranked in the decreasing order of importance. Wuhan, China has been notoriously linked as the birthplace of and received attention in COVID literature [51], [53], [106]. We show in Figure 14 that China hit an early peak by February-March 2020, before other nations (US, Italy, UK) were hard hit by the pandemic. We see a similar peak in the mention of China in COVID-19 literature (Figure 15). From early April 2020, we observe a disproportionately high amount of research within the USA and Italy, while India became a hotspot of COVID research from August onwards. Note that clinical and medical data from Italy and US have been reported in several research articles [107]–[111].

**FIGURE 16.**
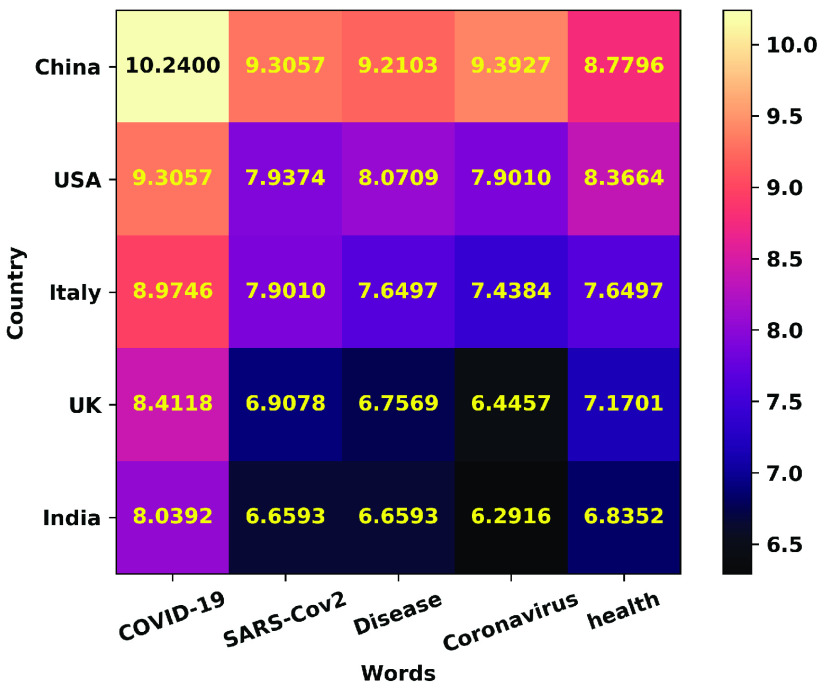
Counts (on a log scale) of the top 5 keywords co-occurring with the top 5 nations mentioned in COVID-19 research abstracts.

**FIGURE 17.**
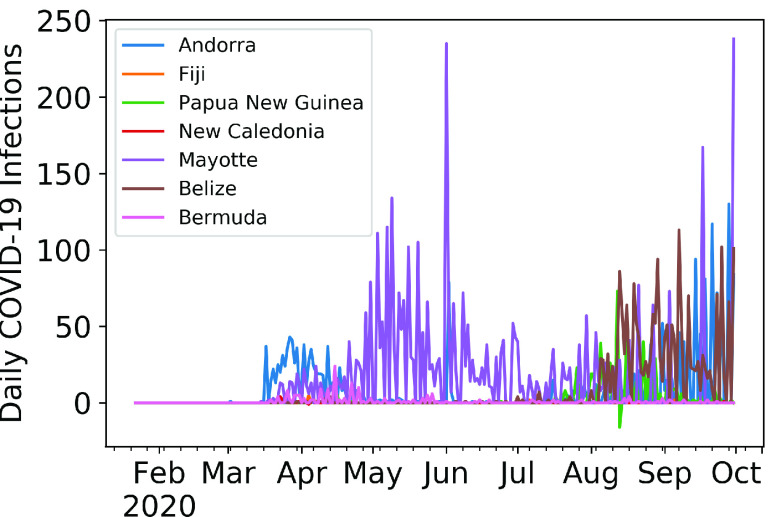
Evolution of daily COVID-19 infection cases in the least mentioned countries.

We next depict the relationship between the top countries and top words. Figure 16 shows the number of mentions of a country alongside the top words on a log scale. China, USA and Italy exhibit the highest presence for the top 5 words, followed by UK and India. From the most mentioned nations, we turn our attention to the least mentioned countries in scientific literature, namely, Andorra, Fiji, Papua New Guinea, New Caledonia, Mayotte, Belize, Bermuda, Kyrgyzstan, and the Northern Mariana Islands (see Figure 17). Andorra comes up rarely in October 2020 in the research [112] in the context of the patient characteristics, ICU mortality factors, and the clinical course of COVID-19 in Spain. Papua New Guinea, Fiji, Northern Mariana Islands, and New Caledonia show up together in March 2020 in [113], evaluating the risks of COVID-19 importation to the Pacific islands.

Mayotte [114] and Vanuatu [115] come up during September - November 2020 and September 2020 respectively, while Bermuda’s cancer care in the COVID era was discussed in [116]. Kyrgyzstan has been discussed at length in genomic research on SARS-CoV-2 in August 2020. Figure 17 shows the daily infection count of the aforementioned nations. These daily COVID-19 infection numbers are exceptionally low (i.e., in the order of hundreds) compared to those from the countries receiving frequent mentions, explaining why they have received less attention from the scientific community.

### D. Identification of Relevant Topics

We apply latent semantic analysis (see Sec. II-C) to identify $d = 10$ topics. We attempted to report some general topics from these four documents and hence, only enlist the top 10 topics; the subsequent topics beyond the top-10 did not represent meaningful and distinctly new directions. We explain in Sec. II-D that each topic is represented as a vector of weights corresponding to the common words. The broad semantic literal meaning can be inferred by analyzing the top 20 words in any given topic, as reported below:

• *Topic 1* $\rightarrow $ COVID-19 Response (top words: COVID-19, Respiratory, Coronavirus, Pandemic)
• *Topic 2* $\rightarrow $ Influenza Response (top words: Influenza, Pandemic, Vaccine)
• *Topic 3* $\rightarrow $ COVID-19 Testing (COVID-19, SARS-CoV-2, Syndrome, Acute, Severe, Positive)
• *Topic 4* $\rightarrow $ COVID-19 Management Strategies (top words: COVID-19, SARS-CoV-2, Wuhan, Management, Strategy)
• *Topic 5* $\rightarrow $> Influenza Response (Influenza, H1N1, Vaccine, H5N1)
• *Topic 6* $\rightarrow $ General Respiratory Syndrome and Coronavirus (top words: Respiratory, MERS-CoV, SARS-CoV, Coronaviruses)
• *Topic 7* $\rightarrow $ Clinical Trial (top words: Admission, Intervention, Dose, Trial)
• *Topic 8* $\rightarrow $ Ebola Response (top words: Ebola, Epidemic, EboV, Africa)
• *Topic 9* $\rightarrow $ COVID-19 Hospitalization (top words: COVID-19, SARS-CoV-2, Surgery, Hospitalization, Admitted)
• *Topic 10* $\rightarrow $ General Hospitalization (top words: Admission, Mortality, Therapy)

**FIGURE 18.**
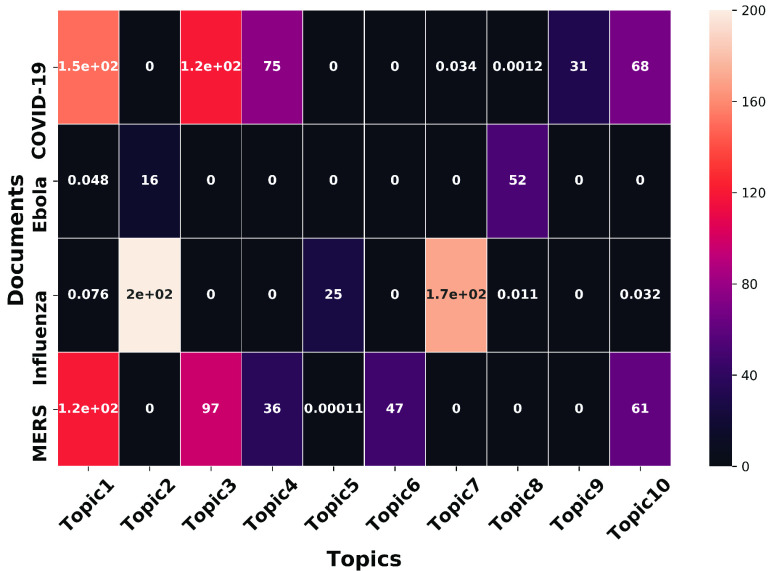
Relationship between disease documents and topics.

Recall that this semantic analysis creates a matrix ($W$) with topic weights contributing to each document. We depict these weights in Figure 18. We observe that COVID-19 research is dominated by topics 1, 3, 4, and 9 dealing with COVID-19 Response, COVID-19 Testing, COVID-19 Management Strategies, and COVID-19 Hospitalization, respectively. We report in Sec. III-E, that MERS shows significant overlap with COVID-19. MERS literature covers general coronavirus, general epidemics, or comparison between virus-borne diseases. On the other hand, topics 2 and 5 contribute heavily towards Influenza, and topic 8 features in Ebola-related publications. Finally topic 7, despite not being disease-specific, has a high weight in the Influenza document. To sum up, 7 out of the 10 topics are mostly seen in COVID-19, Influenza, and Ebola research, while the other 3 cover all four diseases.

### E. Similarity Across Research on Various Diseases

The 250 keywords from the research abstracts on each disease are combined into a list of 403 unique words. Next, we apply the coefficient of variation on the tf-idf scores to rank the words in the integrated list. Recall from our discussion in Sec. II-C that the words with the least CV are the most significant (depicted in Figure 7). We observe that words like *population*, *risk*, *model*, *sample*, *case*, *region*, *vaccine*, *antibody*, *respiratory* and *mortality*, emerge as significant.

**FIGURE 19.**
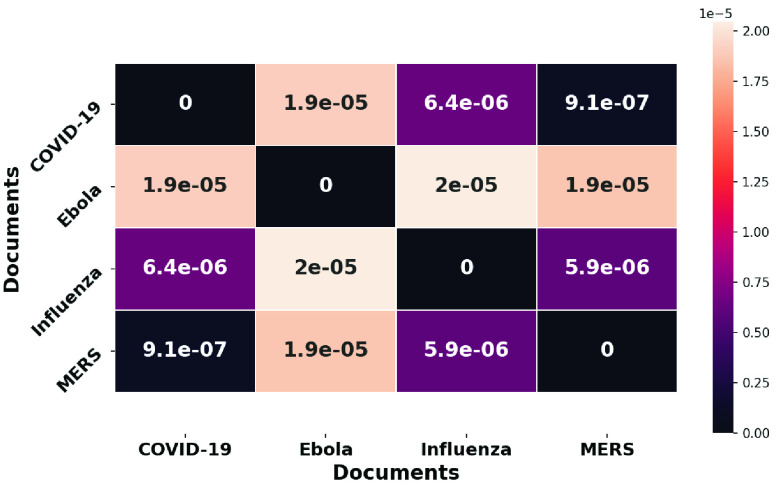
Similarity across disease literature based on mean squared error.

We calculate the mean squared error (MSE) of the tf-idf vectors of 403 common words of the disease pairs and represent the variation among diseases in the form of a heatmap (see Figure 19). Note that MERS and COVID-19 articles are the most similar with MSE $9.1 \times 10^{-7}$, while MERS and Ebola are the most disparate with MSE of $1.9 \times 10^{-5}$. This similarity study reveals the mention of COVID-19 and MERS in the same studies. For example, [14] mentions MERS while talking about the vaccine development strategies for COVID-19 and [117] provides insights into COVID-19 or SARS-CoV-2 in the light of past coronavirus outbreaks like SARS and MERS. Another example of co-occurrence of disease names in the same study is in [67], that showed that H84T has demonstrated antiviral activity against Influenza A and B viruses and can be effective against Ebola. Note that the MSE values are small (of the order of 10^−5^). This is because studies address multiple diseases at the same time [118], [119].

### F. Influential Research Articles

We have gathered the most influential research articles regarding COVID-19 (as discussed in Section II-E). We discuss the first 10 influential research articles as the total number of significant words in the top 10 research articles ranges between 70 and 80. In [120], the success and safety of oral administration of the kinase inhibitor drug was assessed on COVID-19 hospitalized patients. Similarly, the efficacy of antibody test in the diagnosis of COVID-19 in individuals with symptoms for over two weeks and not having an RT-PCR test or having a negative RT-PCR test was reported in [9]. The role of physical intervention in containing spread was analyzed in [121], while [12] performed a controlled trial to gauge the effectiveness of the antiviral activity of hydroxychloroquine against SARS-CoV-2. The ill-effects of tobacco on lung health and respiratory diseases were discussed in [122], and [13] studied the safety of convalescent plasma or hyperimmune immunoglobulin transfusion in the treatment of COVID-19 infected people. Next, an expository article was presented in [61] to help mental healthcare professionals understand the effect of COVID-19 on psychiatric patients, and [10] aimed to judge the diagnostic correctness of point-of-care antigen and molecular-based tests to ascertain whether a person in community, primary or secondary healthcare is COVID-19 infected or not. The evaluation and treatment of coronavirus disease like SARS-CoV, MERS-CoV, etc. was discussed in [123]. Finally, as in [13], [124] evaluated convalescent plasma or hyperimmune immunoglobulin transfusion in treating COVID-19 patients. Since we are still in the early phases of understanding SARS-CoV-2, the majority of the influential articles focus squarely on standard approaches of diagnostics, containment, and treatment.

### G. Implications of the Study

As mentioned in Sec. I-B, the primary goal of our study is to find out the important long-term research topics on COVID-19 in light of the studies on other virus-borne diseases. To assess this, we collected the abstract data on the *long-term impacts* of the respective diseases. Under this search technique, we found 52, 347 and 562 abstracts respectively pertaining to Ebola, Influenza and MERS. This data gives us an idea about the long-term research directions on the other virus-borne diseases. Such works may then imply the probable long-term research directions on COVID-19. We manually identified the dominant research directions from the top-10 topics (that were inferred using latent semantic analysis as before) pertaining to the abstract data on the other diseases (Ebola, Influenza and MERS) which are as follows:

• *Long-term Patient Surveys*
• *Future Disease Outbreak and its Long-term Effects*
• *Intervention Studies*
• *Mental and Other Disease Outcomes*
• *Long-term Vaccination Policies and its Effect on Children and Elderly*
• *Long-term Immunity and its Effect on Patients and Animals*

We next identified the top research directions from the data on the long-term implications of MERS alone as the COVID-19 abstracts were found to be most similar to the MERS abstracts. The broad semantic research areas manually mined from the MERS related abstracts are:

• *Long-term Therapies and Effect on Children*
• *Long-term Patient Surveys*
• *Intervention Studies*
• *Mental and Other Disease Outcomes*

Each of the four long-term research directions align well with our findings mentioned below considering the abstracts from general virus-borne diseases. Mental health, Long-term patient surveys and Intervention studies have emerged as long-term research directions in case of both MERS and the other three virus borne diseases.

First, this study highlights the need for global collaboration to create an international open-access repository that will guide the clinicians, health workers, patients and world leaders to achieve the highest level of patient care. Second, several infectious disease experts, virologists and immunologists agree that COVID-19 will linger as an endemic [125]. Our study shows the merits of designing strategies to curb future outbreaks. Third, our study reveals that the long-term psychological repercussions of COVID-19 surpasses other threats stemming from the virus [126] (see Sec. III-G), making research on COVID-related mental health an absolute imperative. Finally, Fig. 9 shows vaccination to be an under-expressed term in COVID abstracts. This implies that more research will need to go into designing new vaccines to combat new strains of the virus and protecting the elderly, children or patients with preexisting conditions as well as monitoring their effects on overall immunity.

## IV. Limitations of the Study

Our study is based on the research article data collected from PubMed (see Sec. II-A for details). We restricted ourselves to the first 10, 000 entries of each search topic allowed by PubMed. We compensated for this shortcoming by attempting multiple search terms, resulting in minor redundancy in search results. Some results were incomplete with respect to title, author details, publication dates, and contents however, their number was too limited to skew the trends in our findings. Finally, the fact that there is a notable similarity in some disease keywords added to the challenge of sifting through the welter of research articles. Specifically, our initial efforts on exploring SARS as a standalone document in the corpus was rendered impossible as it also picked up SARS-CoV-2 – the virus causing COVID-19.

**Research Gaps in COVID-19:** Our Topic Modeling framework can model some very broad topics related to these four diseases. We identified four topics related to COVID-19 pandemic which were too broad and were unable to identify any topic on social, cultural, economic and psychological impacts of COVID-19. The bulk of research on COVID-19 are on its viral transmission, different medications, clinical trials, etc. but the amount of research on COVID-19 diagnostics, therapeutics, vaccine and genomics are comparatively smaller. Since, the number of COVID-19 infections is still increasing around the globe, there is an urgent need for further research on COVID-19 vaccines, therapeutic measures and diagnostics. The community will also benefit significantly from additional investigations on probable future disease outbreaks. Intervention studies and patient surveys is another important future research topic on COVID-19. Although, there have been some research on mental illness with respect to COVID-19, most of these works fail to bring up the reasons behind this psychological effect.

## V. Conclusion

We performed a comprehensive natural language processing based analysis on the existing scientific literature on COVID-19 to derive keywords that lend social, economic, demographic, psychological, epidemiological, clinical, and medical insights into our understanding of the disease. In light of three diseases, namely, MERS, Ebola, and Influenza, we identify over- and under-represented keywords in COVID-19 research, significant topics, countries, and research articles, as well as their implications on the future of these nascent fields of COVID-19 research.

We use the notion of coefficient of variation to find statistically and semantically important keywords and utilize it to pinpoint trends in the overall science of COVID-19. The references to *healthcare*, *clinical*, *risk*, *morbidity* suggest that the public at large and the scientific community are responding to the immediate threats posed by the virus, and the emphasis of COVID-19 research is squarely on the transmissibility, health implications, short-term treatment plans and public policies (as opposed to long-term immunization and drug-related studies). MERS and COVID-19 exhibit a high co-occurrence in research articles. Topics like COVID-19 Response, COVID-19 Testing, COVID-19 Management Strategies, and COVID-19 Hospitalization forms the majority of the topics in the research abstracts on COVID-19, MERS, Ebola, and Influenza, followed by topics related to Influenza, and then Ebola. No dedicated topic related to MERS has been identified due to the high level of co-occurrence between COVID-19 and MERS-related articles. China, USA, Italy, UK, and India – countries hit hard by the pandemic – have contributed most to scientific studies. The majority of the high-impact articles address questions on diagnostics, containment, and immediate treatment. It is imperative that the researchers continue to leverage their substantial knowledge about SARS-CoV-2 to devise long-term vaccination and drug programs. Particularly, China, the US, UK, Italy, and India can spearhead international projects to create repositories on myriad branches of COVID-19 research.

Our work reports a wide spectrum of insights into the progression of COVID-19 and the course of the associated scientific studies. Comparative analysis between the abstract data on COVID-19 and that on other virus-borne epidemics have shown the need for future research on COVID-19 vaccine, genomics, therapeutics, etc. On top of that, further research on probable future outbreaks, patient surveys and intervention studies will definitely be helpful to the community. Also, we know that psychological problems are well associated with COVID-19 but there are not many works addressing its cause. Hence, it is important to identify whether lockdown or increased viral transmission or other factors are responsible for this illness. Time-dependent topic modeling can also be insightful to understand the temporal patterns in the topics, and hence is an avenue for potential future work.
